# Differential thermal analysis techniques as a tool for preliminary examination of catalyst for combustion

**DOI:** 10.1038/s41598-023-36878-8

**Published:** 2023-06-16

**Authors:** Olena Yurchenko, Hans-Fridtjof Pernau, Laura Engel, Jürgen Wöllenstein

**Affiliations:** 1 Fraunhofer Institute for Physical Measurement Techniques IPM, Georges-Koehler-Allee 301, 79110 Freiburg, Germany; 2 Department of Microsystems Engineering - IMTEK, University of Freiburg, Georges-Koehler-Allee 102, 79110 Freiburg, Germany

**Keywords:** Catalysis, Techniques and instrumentation, Characterization and analytical techniques

## Abstract

The need for more economical catalysts for various combustion reactions is continuously driving catalyst development. We present Differential Thermal Analysis (DTA) and Differential Scanning Calorimetry (DSC) as suitable techniques for fast examination of catalyst activity for combustion reactions. The heat of reaction *ΔH*_*r*_ generated at the catalyst in a combustible atmosphere is the measure for estimating the capability of the catalyst. Present investigations verify the reliability of both methods for the pre-selection of catalysts for further extensive investigations. To simplify the measurements and the result evaluation, a new measurement routine is introduced which is more suitable for rapid catalyst investigation than the conventional approach. For initial investigations, oxidation of 1% methane on a cobalt oxide catalyst was used. First, DTA measurements were performed. The vessel size and the amount of catalyst are considered as factors influencing the thermal signal. Simultaneous mass spectrometry measurements were used to better understand the formation of the DTA response. Comparable DSC investigations were then conducted. Finally, the behavior of catalyst was compared with two commercial palladium/alumina catalysts using DTA and DSC. Our investigations show that DTA and DSC are powerful methods to identify potential catalysts in a fast and reproducible manner, provided that all parameters influencing the thermal signal are kept constant.

## Introduction

Combustion using supported catalysts is utilized in many industrial and environmental applications primarily for fuel reforming or purification, heat energy generation from fuels and to reduce air pollutant emissions from industrial fumes, underground ventilation air in coalmines, etc.^1–7^. The principle of catalytic combustion is also used in some special applications, for example in catalytic gas sensors, in which the heat of reaction gives information about the concentration of a combustible gas^8^. The catalysts revealing more favorable operation conditions, higher conversion efficiency, improved thermal and chemical stability are in demand.

Even small variations in the composition, structure or morphology of the catalyst can provoke a significant change in its catalytic properties^6,9–11^. Consequently, for a comprehensive investigation of catalytic performance, the number of samples can be immense. However, it is desirable that unnecessary time-consuming investigations, which involve always excessive costs, are avoided. To identify potentially interesting catalysts and to reduce the development expenses already at the first stage, different catalyst formulations can be compared qualitatively based on their activity and stability before in the next stage the most promising catalysts are characterized quantitatively regarding their reaction kinetics^12^. For a fast catalyst screening, investigation methods with a straightforward test methodology are favored. In the field of applied catalysis, the catalyst performance for given oxidation reactions is commonly investigated in bed (micro)-reactors by temperature programmed oxidation, for which a detailed analysis of the gas phase is required for reaction monitoring^1–3,6^. Furthermore, the experiments require catalysts in a particular particle size range (40–60 mesh) with a certain porosity to ensure good gas transport through catalyst bed^1,13–15^. For this purpose, the catalysts can be supported, diluted with a further component, pelletized or crushed and sieved to the desired particle size^16,17^. However, the additional preparation step means the introduction of further influencing parameters affecting the catalyst morphology and its catalytic property.

Differential Scanning Calorimetry (DSC) and Differential thermal analysis (DTA) as thermal analysis methods have been also applied for catalytic investigations^18^. DSC is conventionally used to assign variety of thermodynamic quantities of materials, e. g. specific heat capacity *c*_*p*_ or enthalpies *ΔH* of transformations, while the application area of the DTA is the estimation of characteristic transitions temperatures^19^ and the qualitative identification of materials^20^. Beside that firstly DTA^21–23^ and subsequently DSC^24–28^ have been used for the investigation of gas-catalyst interactions including reactants and poisons as well as the catalyst screening by monitoring occurring thermal events. The methods are applicable for the investigation of catalytic processes in which heat is evolved (exothermic reaction) or taken up (endothermic reaction) and preferably a single product is involved. The catalytic combustion as a strongly exothermic reaction is ideally suited for investigating the catalytic activity by the means of thermal analysis methods, especially in case of complete fuel combustion. However, despite many advantages, the methods have not yet been established for catalyst investigations.

The heat of reaction *ΔH*_*r*_ evolved at the catalyst in a combustible gas atmosphere is the basis for determining the catalytic capability and the catalyst screening using DSC and DTA techniques. Two basic types of DSC are distinguished, the heat-flux or heat-flow DSC and the power compensation DSC^19^. The heat-flux DSC, usually termed as DSC in the literature, and the DTA have the same measurement principle, whereby the temperature difference *ΔT* between the sample and an inert reference is measured under non-isothermal conditions (in a scanning mode) while both are heated at a constant rate in a controlled atmosphere. The raw signal obtained by DTA and DSC is the electrical signal in microvolt provided by the thermocouples, whereby the instrumental signal can be converted into the temperature difference *ΔT* or even into the heat value after sophisticated calibration of the detector signal by DSC^29^. The main difference between heat-flux DSC and DTA is the geometry of the sample carrier and sample vessels which results in a higher signal sensitivity in case of DSC^19,20^.

For the determination of the measurement signal related to evolved heat by catalytic reaction, a baseline is recorded in an additional experiment at the same thermal conditions, but in an inert atmosphere^23^. However, the baseline measurements in an extra experiment is the most critical issue due to low reproducibility making these measurements more challenging^30,31^. Furthermore, the result interpretation and evaluation of the catalytic activity from experiments performed in a scanning mode at non-isothermal conditions is quite sophisticated and less appropriate for the intended rapid catalyst screening. DTA method is even less applicable for catalytic investigations in a scanning mode. The measurements performed at isothermal conditions are easier to interpret in terms of catalytic activity, although the difficulties encountered with the baseline recording are not resolved^28^. The further advantage of operation at isothermal mode is that the measurements are performed at steady-state conditions with defined temperature gradient which is important for consistent results^23^.

In our view, the difficulties associated with the baseline measurement can be overcome if the baseline and the experimental data are recorded within the same experiment. This becomes possible when the isothermal measurement mode is used, and the air and the examined combustible gas are introduced successively into the chamber. This procedure is usually used for the testing of gas sensors and additionally has the advantage for DTA and DSC that small external thermal fluctuations or thermal gradients emerging in the system during the heating are excluded. Thermal fluctuations due to gas flow variations have only insignificant effect on the measured signal. Such a simplified measurement procedure is suitable for DTA and DSC investigations and is more convenient for fast catalyst preselection than the standard procedure. Since the goal of investigations is the choice of potential catalysts and not the estimation of kinetic or thermodynamic parameters, the relative temperature-dependent Seebeck-voltage of the thermocouples is quite sufficient for the comparison of catalyst activity. A complex calibration of detector concerning heat flow, in order to convert the voltage signal in the energy signal (mW) is therefore no longer required. It is a big advantage, because the calibration of DSC signal, and of DTA signal even more, is difficult in case of gas–solid reactions. The calibration should be performed with a closed vessel, whereas the gas–solid experiments are carried out with an open vessel effectuating that due to thermal losses evolved gas analysis is necessary to obtain the calibration constant^27^.

In previous papers^32–34^, we have already reported about the applicability of the DTA method for catalyst investigations. We evaluated the effect of particle size and morphology of a cobalt oxide catalyst on its thermal response to methane oxidation^32^ and examined its thermal stability in methane containing atmosphere using DTA^33^. It could be shown that different catalysts can be tested based on a comparison of their thermal response^34^. The main motivation for the investigation was the need for new catalysts for catalytic gas sensors^35^.

In the present paper we show that the applied investigation procedure is well suited for the catalyst examination by means of DTA as well as DSC. Methane (CH_4_) as a main component of natural gas and as a byproduct of many human activities^1^ takes an important role in application of catalytic combustion and for the monitoring of combustible gases by catalytic gas sensors^36^. CH_4_ has been therefore chosen as test gas. The basis experimental investigations were performed on spinel cobalt oxide (Co_3_O_4_) catalyst because previous works showed its high stability which is important for correct method evaluation. Moreover, Co_3_O_4_ with variable valence states (Co^2+^/Co^3+^) has attracted increasing attention as a very active non-noble metal catalyst for the catalytic combustion of CH_4_^2,10^ or as a support for noble metal catalyst^37^. With the focus on the sensor application, the temperature range between 250 and 450 °C was chosen for the investigations. To ensure the reliability of the DTA and DSC method for the investigation of catalyst activities, theoretical considerations are first made on thermal signal formation identifying fundamental influencing parameters. Subsequently, DTA experiments are performed on Co_3_O_4_ in combination with simultaneous quadrupole mass spectrometry (MS) measurements under exposure to 1% methane in dry air. MS measurements of the evolved gas as a direct measure of catalytic reaction are carried out for verification and a better understanding of thermal signal formation. In experiments, the impact of two instrumental parameters, the crucible size or geometry and the amount of catalyst used for measurements was examined and their influence assessed. Accordingly, the differences observed in MS and DTA signal were discussed considering the influencing factors. Afterwards, similar DSC experiments with different amount of catalyst were carried out to demonstrate the particularities of the method. Finally, the prospects for the application of DTA and DSC are illustrated by a comparison of the thermal behavior of a Co_3_O_4_ catalyst with that of two commercial Palladium/alumina (Pd/Al_2_O_3_) catalysts.

## Methods

### Materials and characterization methods

The commercially available STA-QMS (NETZSCH, STA 409 CD-QMS 403/5 SKIMMER) system equipped with an interchangeable DTA or DSC sample carrier with integrated thermocouples (type S) was used for investigation of the catalytic activity at dry conditions. The temperature sensor of the furnace was calibrated by melting of metals using a standard procedure^23,29,38^. Dry synthetic air was applied for the recording of the baseline and a pre-made mixture of 1 vol% CH_4_ in dry synthetic air was used as test gas (Air Liquid, Germany). The experiments were performed at a constant gas pressure of 0.5 bar and a gas flow rate of 100 mL min^−1^ regulated by computer-driven mass flow controllers under isothermal conditions within the temperature range of 250 < T < 450 °C. Thermal stability experiments were performed using compressed dry air containing 400 ppm CO_2_ (dew point < − 20 °C).

**Figure 1:**
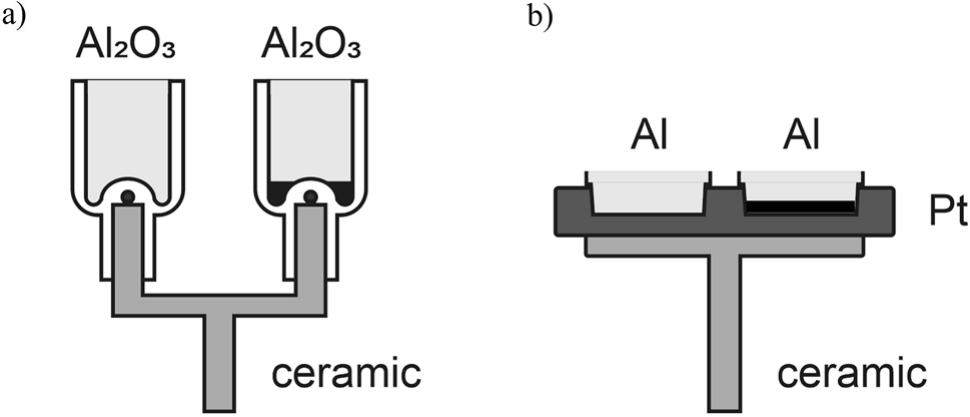
Schematic view of DTA (**a**) and DSC (**b**) sample carriers. DTA ceramic carrier holds aluminum oxide reference (left) and sample (right) vessels in form of a crucible. DSC ceramic carrier containing platinum platform with thermocouples holds aluminum pan for reference (left) and sample (right).

The ceramic sample carriers and sample vessels used for DTA and DSC measurements differ in the structure and form (Fig. 1). In DTA sample carrier, the crucibles, have a point contact and in DSC, the pans, the surface contact via platinum platform with the thermocouples. Thermocouples enable the measurement of the temperature directly at sample and reference crucible or pan. Aluminum oxide crucibles were chosen for DTA experiments due to their inertness. Two sizes of crucibles were available, the “flat” crucible (vessel height 10 mm, total height 16 mm) denoted as *fc* and the “deep” crucible (vessel height 13 mm, total height 23 mm) denoted as *dc*. Both crucibles have an inner diameter of 6 mm and thus the same area for interaction with the gas. With 1127 mg and 1455 mg, they differ in mass. For DSC measurements, the aluminum pans (vessel height 2 mm, inner diameter 5 mm, mass approximately 23.5 mg) denoted as *pn* were used due to good thermal conductivity of aluminum. In both cases, an empty vessel was used as reference. The experiments were conducted with open vessels to allow gas exchange.

Initial investigations to validate thermal analysis were performed using DTA method coupled with MS investigations. DSC measurements were conducted without simultaneous MS measurements as the platinum platform of DSC sample carrier shows a certain catalytic activity in oxidation reactions. However, the catalytic activity of platinum is not of significant importance for thermal analysis due to symmetry of the sample carrier and similar temperature on both vessels. Co_3_O_4_ synthesized by precipitation procedure as already described elsewhere was applied as catalyst^32^. Co_3_O_4_ catalyst was used owing to its high stability which is of crucial importance for correct method verification. To investigate the impact of the catalyst mass, a powdered Co_3_O_4_ catalyst with a mass between 8 and 20 mg or 4 and 16 mg was used for DTA and DSC measurements, respectively. Powder samples enable a good contact with the bottom of the vessel resulting in good heat transfer.

In subsequent DTA and DSC experiments, two commercial initially reduced aluminum oxide supported palladium catalysts with 10 wt% (Aldrich, Germany) or 5 wt% (Acros Organics) Pd loading termed as c-10Pd/Al_2_O_3_ and c-5Pd/Al_2_O_3_ were used to compare the thermal behavior of the Co_3_O_4_ catalyst. The DTA experiments were performed in *dc* crucibles with 20 mg powder, while 8 mg of catalysts were used for DSC measurements. Different catalyst mass was chosen for DTA and DSC examinations due to unlike size and volume of the vessels. The optimal mass of a sample for DTA and DSC vessels is therefore different.

The investigated catalysts were characterized by scanning electron microscopy (SEM) and energy dispersive X-ray (EDX) spectroscopy using SU-70 (Hitachi High-Tech Corporation).

### System description

**Figure 2:**
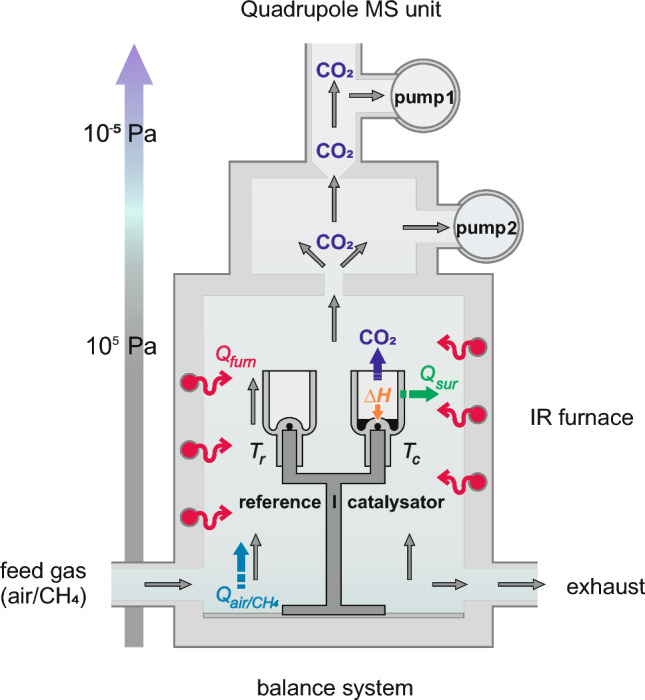
Scheme of STA/QMS with DTA sample carrier showing gas streams and heat distribution.

The used STA 409 CD + Skimmer combines thermogravimetry (TG) technique with thermal analysis techniques, thereby DTA or DSC sample carrier can be applied. Additionally, a quadrupole mass spectrometer is coupled with the TG-Thermal Analysis system, allowing simultaneous analysis of gases produced during the experiment down to the ppm range. Figure 2 shows a schematic illustration of the measurement setup using DTA sample carrier.

In TG–DTA (Fig. 2) (or TG-DSC), the sample carrier balance system enables the measurement of the temperature along with the mass change of the sample. The TG signal is not considered in these investigations, although TG can be used for investigations of certain catalyst^39–41^. The primary measurement signal *ΔU* provided by the thermocouples is proportional to the temperature difference *ΔT* between sample and reference due to the catalytic oxidation of methane and is given in µV. The signal can be converted into µV mg^-1^ by relation to the known catalyst mass allowing the direct comparison of catalysts activity. Due to the heat release during catalytic oxidation, thermal signals show a negative output. The required thermal conditions are adjusted by an infrared heating spiral, which is integrated in the furnace shell. The experiments are performed under a constant gas flow which supplies/removes reactants to/from the catalyst surface and the produced gases to the mass spectrometer. The gases are introduced into the system in the lower part of chamber and heat up before they reach the catalyst in the crucible. A part of the initial gas stream flows directly into the exhaust, including some of the gas released. The other part of the gas stream is passed through two pumping stages that deliver the pressure drop from 10^5^ to 10^–5^ Pa into the MS unit. In MS unit, the gas molecules are ionized, and the formed gaseous ions are successive analyzed by their mass/charge (m/e) ratio at certain intervals. The ion current of 44 m/e, which is characteristic for the oxidation product CO_2_, was analyzed as a part of the experiments.

#### Methodology

The following considerations are performed on the example of DTA method, but they are valid for DSC as well. The specifics of DSC will be indicated. In DTA experiments (Fig. 2), the sample and the empty reference crucible are heated in gas flow to the certain temperature. At a defined temperature, the chamber is first flooded with air for baseline construction and subsequently with a CH_4_ mixture. At isothermal conditions (*T*_chamber_ = const) in air atmosphere, the temperature of reference *T*_*r*_*(air)* and the catalyst crucible *T*_*c*_*(air)* is in thermal equilibrium (Eq. 1). This equilibrium is adjusted by constant negligible IR radiation from furnace *Q*_furn_ and constant heat dissipation *Q*_air_ by air flushing continually through the chamber.

1 $$ T_{r} \left( {air} \right)\mathop \leftrightarrow \limits^{{Q_{furn} , Q_{air} }} T_{c} \;({\text{air}}),\;\;\; T_{chamber} = const $$

The DTA signal in air serves as a baseline for the signal recorded in CH_4_ containing atmosphere. This routine helps to compensate any possible inequalities of the system caused by slight differences in the heat distribution or temperature gradients inside chamber that occur.

The catalytic experiments take place under a complete oxidation of methane to CO_2_, with an excess of oxygen* p*_*O2*_ >  > *p*_*CH4*_, according to Eq. 2.

2 $${CH}_{4}+2{O}_{2}\stackrel{T, {p}_{O2, } {P}_{CH4} }{\to }= {CO}_{2}+2{H}_{2}O, {\Delta }_{c}{H}^{^\circ }=-\mathrm{890,8 kJ}/\mathrm{mol}$$

The heat *ΔH*_r_ produced on the catalyst surface owing to CH_4_ oxidation depends mainly on the standard heat of methane combustion *Δ*_c_*H°* (thermodynamics) and additionally on the extent of reaction or reaction rate (kinetics). It is important to mention that the signal obtained by thermal analysis corresponds to reaction rate and gives no information about conversion efficiency. Higher reaction rate causes higher measured thermal signal. This is the main difference to other investigation methods which examine the conversion efficiency (in %) at given conditions.

Due to the heterogeneous reaction, the reaction rate depends on the temperature and the catalyst surface coverage Θ with reactants which in turn is influenced by the gas partial pressure, *p*_*O2*_ and *p*_*CH4*_, or in other words by the gas concentration^42^. Especially the dissociative chemisorption of CH_4_ molecules needs activation which is associated with high activation energy ${E}_{a}$. The reaction proceeds to a sufficient extent when the operation temperature *T* is high enough to overcome the activation energy barrier ${E}_{a}$. Therefore, the operation temperature *T* is one of the experimental variables of the intended investigations, which characterizes the catalyst behavior. The partial pressures *p*_*O2*_ and *p*_*CH4*_ are kept constant during experiments.

If the CH_4_/air mixture is introduced into the chamber instead of air at constant furnace temperature *T*_*furnace*_ and the heat *ΔH*_r_ is released on catalyst, a new thermal equilibrium is established between reference crucible with *T*_r_(CH_4_) and catalyst containing crucible with *T*_c_(CH_4_).

3 $$ T_{r} \left( {CH_{4} } \right)\mathop \leftrightarrow \limits^{{Q_{furn} , Q_{CH4/air} }} T_{c} (CH_{4} ) = T_{r} (CH_{4} ) + \Delta T, T_{chamber} = const $$

Part of the released heat *ΔH*_r_ is transferred to the crucible through the catalyst powder increasing its temperature *T*_c_(CH_4_). Another part of the heat *ΔH*_r_ is lost for detection due to its dissipation into surrounding *Q*_*sur*_^27,28^. The temperature difference between reference and catalyst *ΔT* (Eq. 4) is determined, besides the heat produced during reaction *ΔH*_r_, by specific heat capacity of catalyst *c*_*p(c)*_ and vessel *c*_*p(ves)*_ effecting the heat storage, and the heat loss to the surrounding *Q*_*sur*_. We expect that the most of catalysts to be investigated have comparable heat capacity* c*_*p(c)*_, which allows the comparison of activity of variable catalysts.

4 $$\Delta T=f(\Delta {H}_{r}{, c}_{p\left(c\right)}, {c}_{p\left(ves\right)}, {Q}_{sur},{{ m}_{c}, m}_{ves})$$

The heat loss to the surrounding *Q*_*sur*_ is mainly due to heat convection from the gas flow. The higher heat flux to the surrounding is in turn expected at higher temperature difference to the surrounding, which should lead to a lower effective thermal signal at higher *ΔH*_r_. As observed by preliminary investigations, the gas flow rate $\dot{V}$ has a considerable impact on the transfer of reactants or products (CH_4_, formed CO_2_) to and from the catalyst surface and consequently on thermal signal. For this reason, $\dot{V}$ is kept constant at 100 mL min^−1^ for all experiments.

The mass of the catalyst *m*_*c*_ as a measure of quantity effects directly the heat produced during the reaction *ΔH*_r_. Furthermore, the geometry and the mass of vessels should have a considerable influence on the measured signal *ΔT*. The geometry affects the mass transfer of gases and heat distribution, while the mass *m*_ves_ along with the heat capacity *c*_*p(ves)*_ effect the amount of heat accumulated in the vessel. It should be noted that the mass of DTA crucible *m*_ves_ is up to ten times higher than the mass of catalyst *m*_c_. For DSC, *m*_ves_ and *m*_*c*_, are in the same order implying lower heat losses to the pan. Additionally, the geometry of DSC pan and sample carrier enables higher mass transfer of gases and better heat transport to the thermocouples across the surface between the pan and the platinum crucible bed. Moreover, the thermal conductivity of aluminum (235 Wm^−1^ K^-1^ at 20 °C) is ten times higher than of aluminum oxide (12–30 Wm^−1^ K^−1^). These features ensure higher sensitivity and faster steady state for the DSC method compared to DTA. However, Al pans possess lower thermal and chemical stability than Al_2_O_3_ crucibles and cannot be used in some specific experiments, e.g. catalyst poisoning.

Simultaneous to DTA, the MS signal of formed CO_2_ will be measured. In contrast to DTA signal, the CO_2_ signal obtained from the mass spectrometer should directly reflect the methane conversion and thus provides information about catalytic activity, since it does not depend on thermal properties of the system components. However, the reactant transport should influence the signal formation. The quantitative statements are only possible after calibration of the MS signal with CO_2_ gas mixtures with different gas concentration using a special device unit. In the investigations shown, the DTA signal is compared with an unquantified MS signal.

## Results and discussion

### Catalyst characterization

**Figure 3:**
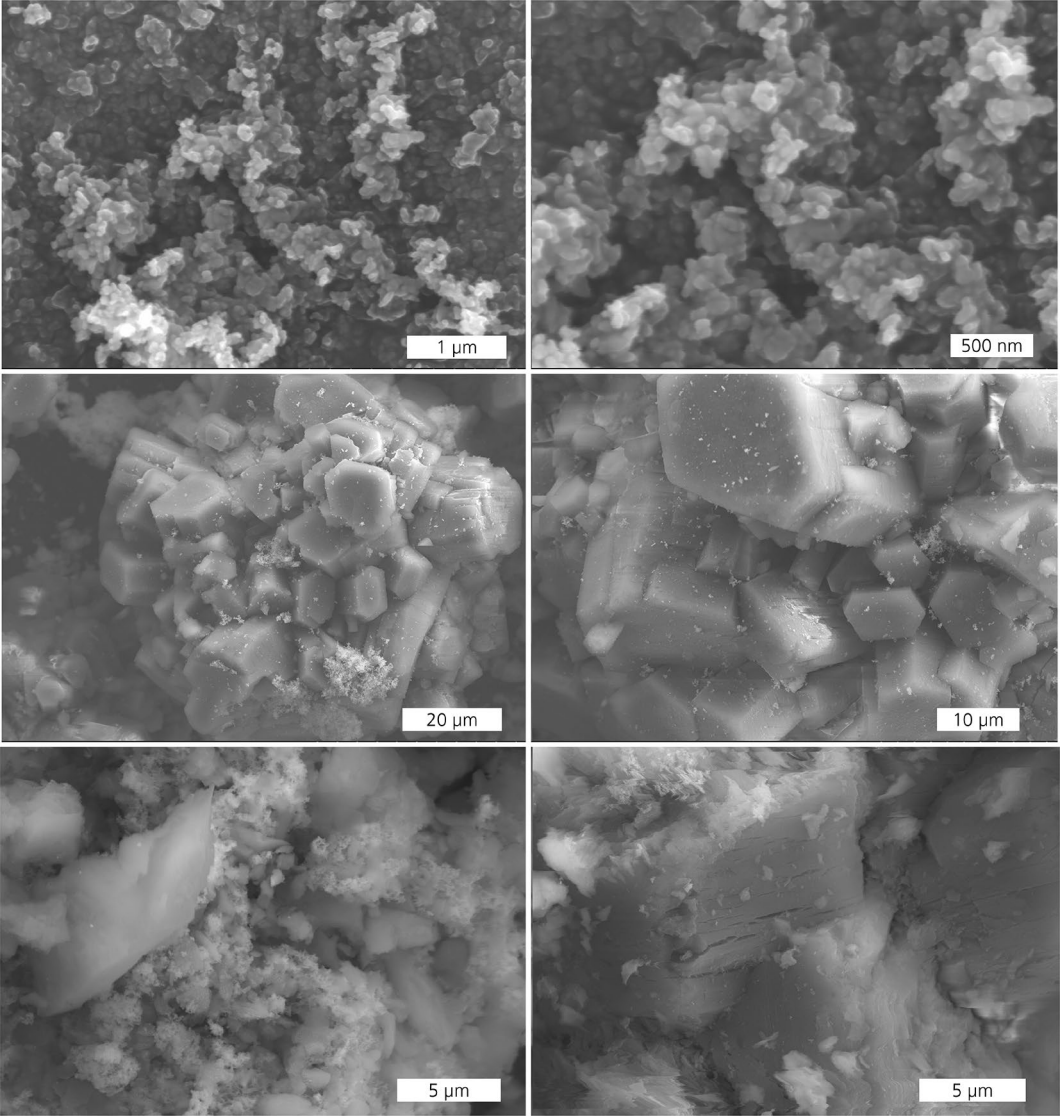
SEM micrographs of Co_3_O_4_ (top) c-5Pd/Al_2_O_3_ (middle) and c-10Pd/Al_2_O_3_ (bottom).

The SEM images of catalysts used for the investigations are shown in Fig. 3. Co_3_O_4_ (Fig. 3, top) exhibits small nanoparticulate particles of a non-spherical shape. The average particle size does not exceed 250 nm. Al_2_O_3_ in c-5Pd/Al_2_O_3_ (Fig. 3, middle) exhibits micrometer-sized crystals of a hexagonal structure. Pd particles are distributed over the alumina surface or accumulated in crystal interspaces as agglomerates. According to EDX the average Pd content for the area investigated was 4.2 wt% (Supplementary Information, Fig. 1, Table 1), whereby the Pd distribution in the catalyst is not very homogeneous owing to the Pd agglomerates.

Otherwise, Al_2_O_3_ in c-10Pd/Al_2_O_3_ (Fig. 3, bottom) reveals particles of not defined structure and morphology and varied particle size in micrometer range. Pd particles mainly exist in the form of aggregates which are not homogeneously distributed in the catalyst. The not homogeneous particle distribution is affirmed by the different average content of Pd (34.2 wt% and 12.9 wt%) found by the EDX measurements (Supplementary Information, Fig. 2, Table 2).

### Thermal analysis

The thermal signal measured by DTA and DSC experiments is a relative and not an absolute value, the magnitude of which depends on different instrumental parameters. The initial experiments were performed by DTA-MS to examine the basic conditions for the application of thermal methods in catalyst investigation. The effect of vessel geometry or mass *m*_ves_ as well as the influence of the catalyst mass *m*_c_ on the DTA signal has been investigated at a fixed gas flow rate $\dot{{\varvec{V}}}$. The MS signal is measured to gain further insights into the DTA method. Next, DSC measurements were performed with different catalyst mass *m*_c_. Finally, some examples for evaluating the catalyst activity are presented.

#### Evaluation of the effects of instrumental parameters

**Figure 4:**
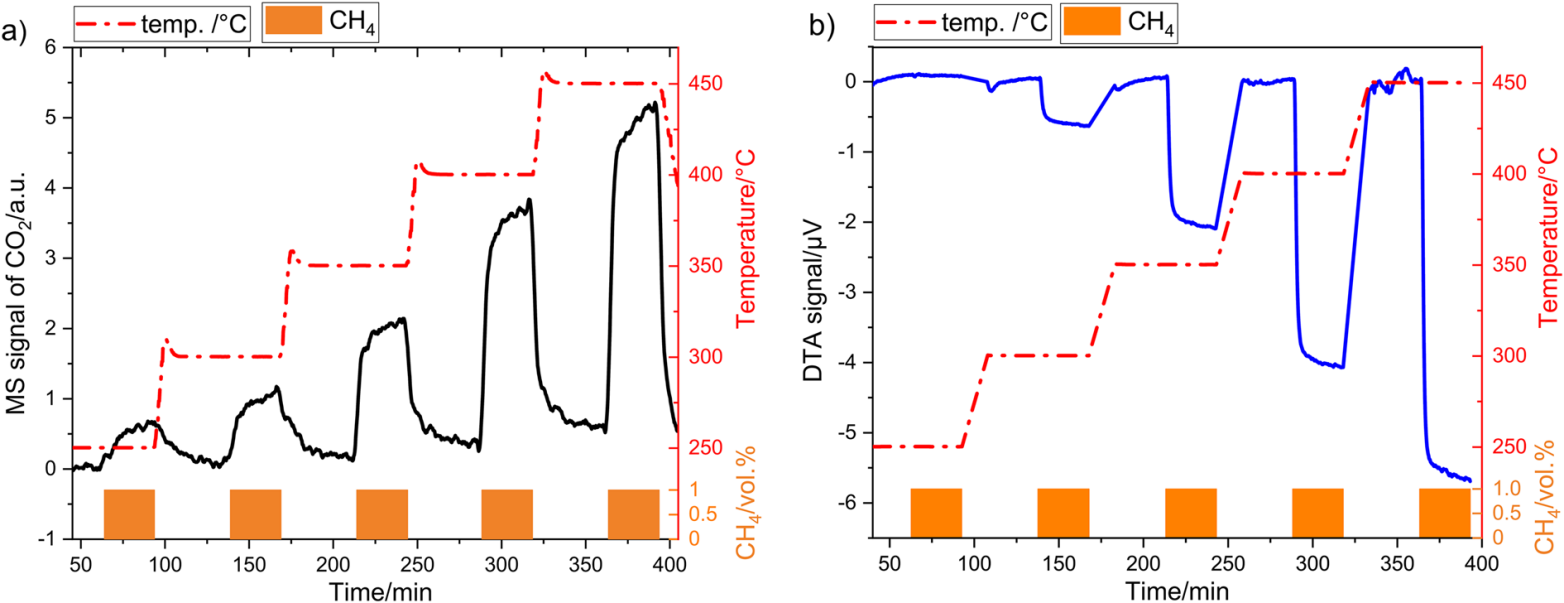
(**a**) MS signal of CO_2_ formed during the combustion reaction of 1 vol% CH_4_ and (**b**) the corresponding DTA signal in the temperature range between 250 and 450 °C. Co_3_O_4_ powder (8 mg) in a "flat" pan was used as a catalyst.

Figure 4 illustrates exemplarily the evolution of (a) MS and (b) DTA signals observed with 8 mg Co_3_O_4_ catalyst in *fc* crucible upon exposure to 1 vol% methane (orange bars) at a predefined temperature program (red line). It should be noted that the baseline of MS signal in air (Fig. 4a) shifts steadily to a higher ion current during the experiment, which could be attributed to the activity variation of the ion source under experimental conditions. This circumstance means that MS signals can only be determined with a certain error. To build-up a baseline of DTA signal (Fig. 4b), signal recorded in air was set to zero at each temperature level. In addition, only isothermal segments are shown, while segments recorded during the heating are cut out.

**Figure 5:**
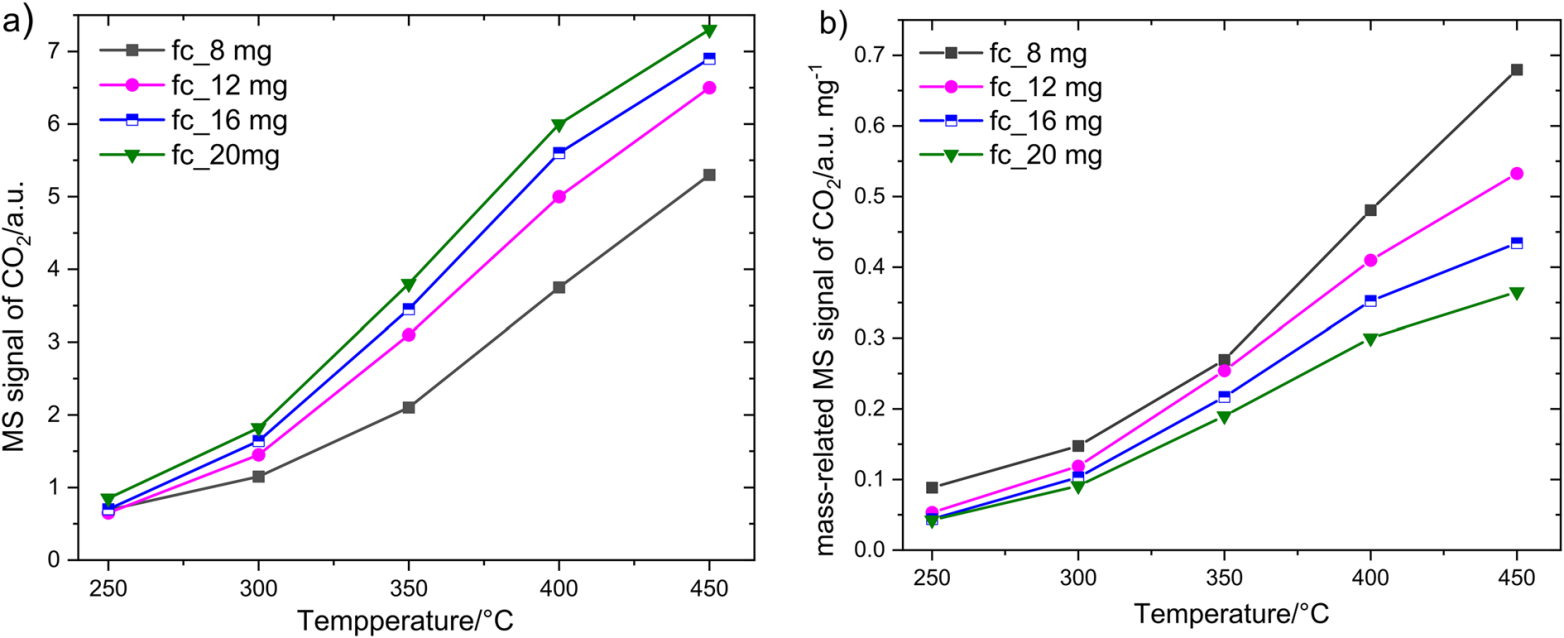
Temperature dependence of the MS signal obtained for different amounts of Co_3_O_4_ catalyst using a “flat” crucible (**a**) without and (**b**) after relation to catalyst mass.

Both MS and DTA curves show an initial signal increase upon exposure to methane followed by a signal stabilization at a certain value which corresponds to steady state at given conditions (Fig. 4). It is obvious that both methods show a different signal development with increasing temperature, which results from the peculiarities of these methods. The following investigations consider the influence of the crucible geometry and the amount of catalyst on the development of both signals. Firstly, the MS signal of CO_2_ formed during catalytic methane oxidation is considered. Figure 5 shows the MS response of 8, 12, 16 and 20 mg Co_3_O_4_ in dependency on the temperature measured in a *f*_*c*_ crucible. For comparison, the total MS signal (Fig. 5a) was related to the catalyst amount (Fig. 5b). A sufficiently large MS signal (Fig. 5a) is already observed at 250 °C. The rise in reaction temperature is associated with an increase of the CO_2_ signal correlating with a higher reaction rate. The increase in catalyst mass also leads to a higher MS signal. When MS signal is related to the mass of the catalyst (Fig. 5b), it is visible that a lower amount of catalyst reveals a higher mass normed response than the higher amount over the entire investigated temperature range. This indicates that with a higher catalyst mass, the CH_4_ transport within the catalyst layer is limited by diffusion and/or the accessibility of the deep laying catalyst particles for reactants declines. Diffusion limitations effect the appearance of concentration gradient of reactants inside the catalyst layer which causes the lowering the surface coverage Θ of the deeper laying catalyst particles with CH_4_ molecules. This reduces the overall reaction rate.

**Figure 6:**
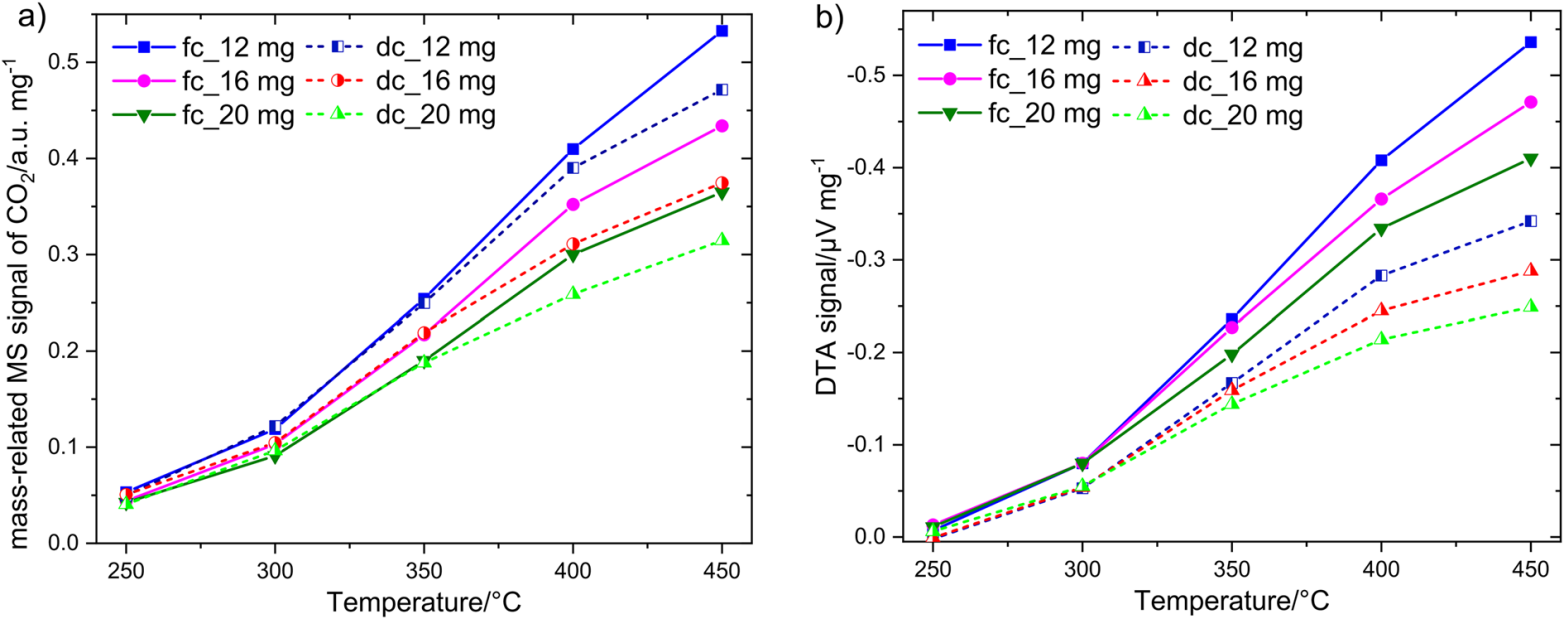
Comparison of (**a**) the catalyst mass-related MS signal and (**b**) the catalyst mass-related DTA response obtained for different amounts of Co_3_O_4_ catalyst using “flat” and “deep” crucibles in 1% CH_4_ as function of temperature.

The effect of temperature on the catalyst mass-related MS signal with varying amount of catalyst is shown for both types of DTA crucible in Fig. 6a. The MS signals for *fc* and *dc* are similar up to 350 °C, which is expected, if experiments are performed at the same conditions (temperature, catalyst amount and equal contact area with the gas). Latter is ensured since both vessels have equal diameter. However, at higher temperatures with increased methane conversion the MS signal obtained for *fc* crucibles (solid lines) is larger than that for corresponding catalyst masses in *dc* crucibles (dashed lines). Such behavior suggests that product removal from the crucible into the gas phase and their transport to MS unit is more restricted for the “deep” crucible due to its higher walls.

The respective data obtained from simultaneous DTA measurement are shown in Fig. 6b. In contrast to the MS measurements, in which a clear mass-related signal is already observed at 250 °C (Fig. 6a), a hardly noticeable DTA response is obtained at this temperature (Fig. 6b). The increasing the temperature and catalyst mass *m*_*c*_ increases the DTA response for both crucibles. However, it is visible that the pronouncement of the signal depends stronger on the crucible type than for MS signal. The *fc* crucible shows obviously a higher response for the same *m*_*c*_ at all temperatures. The higher sensitivity of a “flat” crucible can be explained by lower mass of crucible *m*_*ves*_ (1127 mg vs. 1455 mg). Thus, the amount of heat stored in *fc* crucible is lower and the *ΔT* reached at steady state at the thermocouple is higher. The difference between the MS and DTA signal at 250 °C is a result of the heat dissipation on the crucibles. The heat generated during catalytic oxidation is not sufficient to heat the crucible and to induce a pronounced thermal signal.

The next interesting observation is that both crucibles exhibit a similar signal height for entire range of *m*_*c*_ at 250 and 300 °C, while the respective signals drift apart with increasing temperature. The non-dependence of the signal on *m*_*c*_ at 250 °C and 300 °C evidences the kinetic reaction regime without any diffusion limitations. If the difference in signal arises and becomes larger for different catalyst mass, this is a sign of an increased diffusion limitation of methane within the catalyst layer and/or inside crucible and thus a lower reaction rate as without any limitations^43^. This conclusion is in line with the catalyst mass-related MS signal (Fig. 6a). The results exhibit the different signal sensitivity for “deep” and “flat” crucibles which originates from differences in their geometry and thermal mass. Despite different signal sensitivity, the mass *m*_*c*_ and the temperature dependence of the DTA signal are similar for both DTA vessel types. This evidences that both vessels can be used for catalytic investigations on condition that the same catalyst mass is used for examinations.

**Figure 7:**
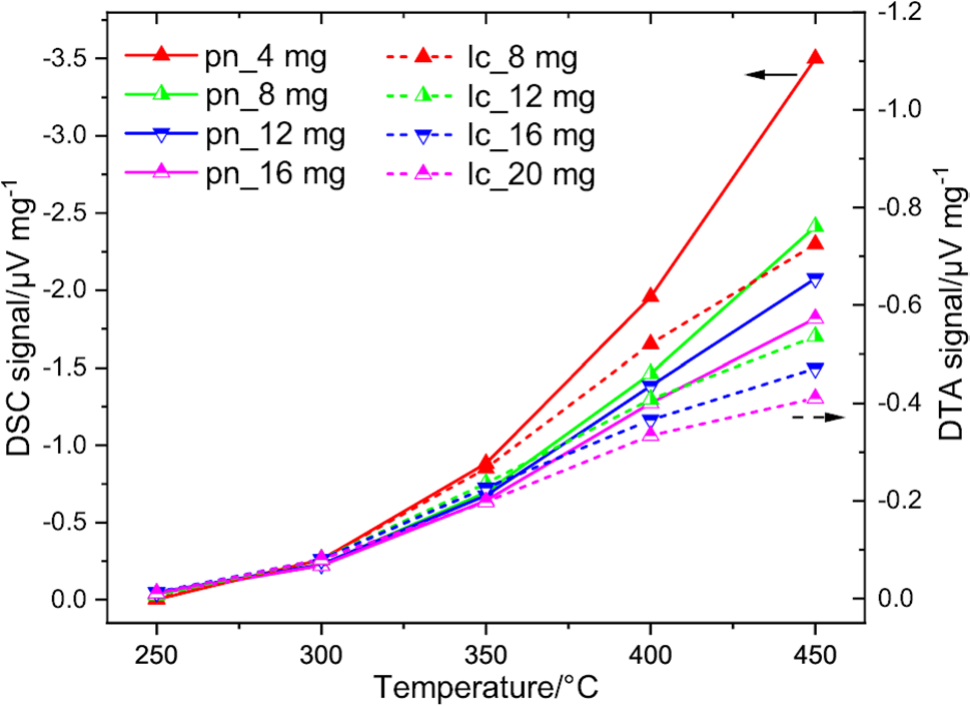
Comparison of the catalyst mass-related DTA (dashed line, scale right) and DSC (solid line, scale left) responses for different amounts of Co_3_O_4_ to 1% CH_4_ as a function of temperature.

Next, the DTA signal related to the catalyst mass, measured in “flat” crucible, is compared with the DSC signal for different amounts of catalyst. The catalyst amount used for DSC measurements was 4, 8, 12 and 16 mg due to the smaller size of the DSC pan. As expected from considerations in methodology part, DSC reveals considerably higher signal sensitivity than DTA (Fig. 7), although the dependence of thermal signal on the temperature for different catalyst mass *m*_c_ is similar. One can see almost no signal at 250 °C and a marginal signal at 300 °C. With further increasing of the temperature the signal respectively increases and the signal values diverge for different *m*_c_ referring to diffusion limitations. Since the DSC pan is 2 mm high, the diffusion limitations are attributed to the methane concentration gradient inside the catalyst layer, while in DTA crucibles the additional methane transport limitations inside the crucible emerge. The results of DTA and DSC measurements indicates that as soon as multiple catalyst layers are used, the diffusion limitations inside the layer appear. This factor can be considered by using of lower *m*_c_.

In summary, it can be said that the same catalyst mass must always be used in experiments to correctly estimate and compare the catalytic activity of different catalysts. The same recommendation were made by Loskyll *et al.*^28^. Indeed, very low and very high catalyst quantities should be avoided to ensure a uniform distribution of the catalyst particles in the vessel. DSC reveals higher thermal responses at identical measurement conditions than DTA owing to the different geometry of the sample carrier and the vessel. Although the absolute signal values obtained with two DTA crucibles und DSC pan are different, thermal signals show similar temperature dependence. The higher sensitivity of DSC or DTA with *fc* crucible can be advantageous for reactions with low reaction enthalpy $\Delta {H}_{C}^{^\circ }$, e.g. CO oxidation. It allows also a more precise differentiation of the catalytic activity of different catalysts. Further DTA investigations were performed using “deep” crucible with 20 mg catalyst, whereas for DSC examinations 8 mg catalyst were used.

### Application example: characterization of signal reproducibility and thermal stability

To demonstrate the potential of thermal methods for catalyst characterization, Co_3_O_4_ and commercial Al_2_O_3_ based catalysts with different Pd content, c-10Pd/Al_2_O_3_ and c-5Pd/Al_2_O_3_, were investigated in relation to signal height, reproducibility and their thermal stability. The catalyst with higher Pd-loading should exhibit increased catalytic activity and higher thermal signal.

Signal reproducibility is estimated by consideration of standard deviation σ of the measured value x_i_ from the mean value µ according to Eqs. (5, 6)

5 $$\mu = \sqrt{\frac{1}{N} \sum_{i=1}^{N}{x}_{i}}$$

6 $$\sigma = \sqrt{\frac{1}{N} \sum_{i=1}^{N}{({x}_{i}-\mu )}^{2}}$$

where N is the number of measurements in a set.

By interpretation of data, the accuracy of the measurements should be considered. The accuracy of the DTA and DSC measurements is strongly influenced by the preparation of samples. Based on obtained results, the measurement uncertainly for different samples is expected to be between 1 and 5% and for the same sample < 3% provided that the properties of the sample remain over time unaffected. In case of higher signal deviations, other underlying causes must be assumed.

**Figure 8:**
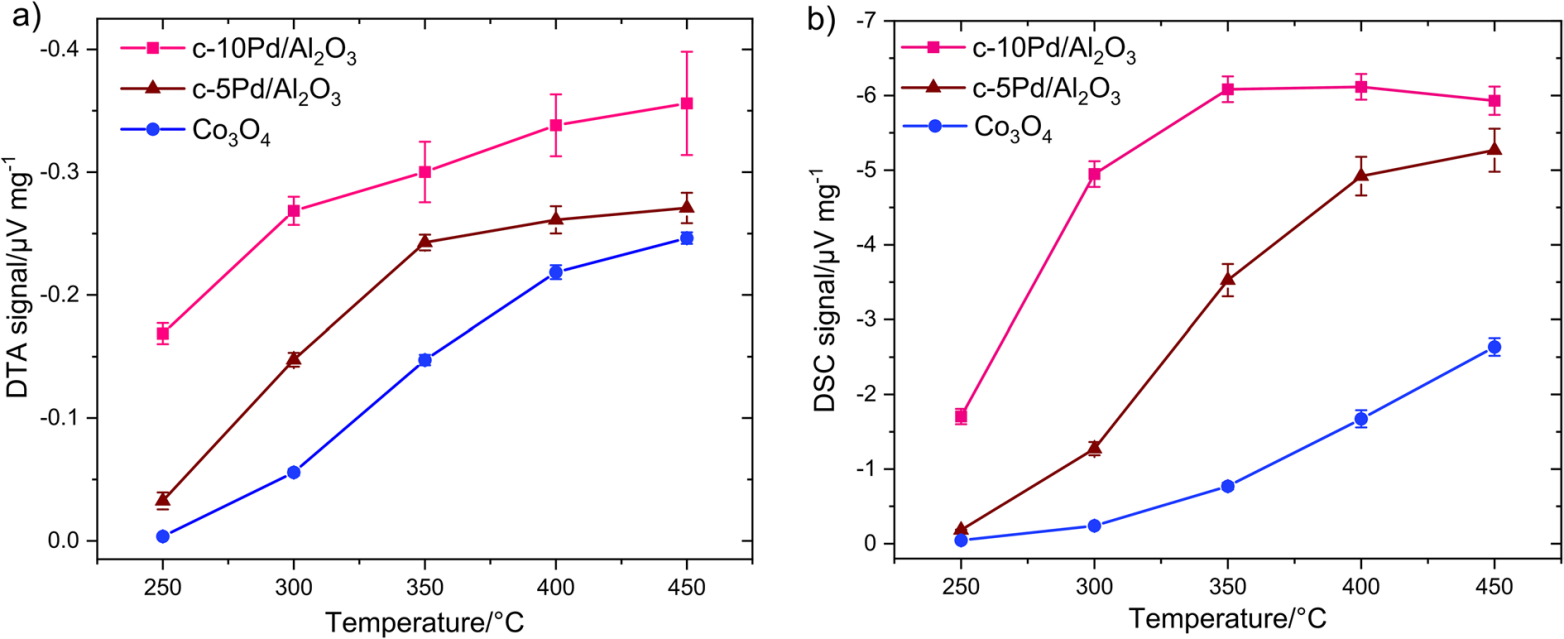
Temperature dependent response to 1% CH_4_ along with error bars measured for three catalysts using (**a**) DTA and (**b**) DSC. There are 6 months between DTA and DSC measurements.

Figure 8 shows the temperature dependence of the mean value of (a) DTA and (b) DSC response for three catalysts with the respective standard deviation. When comparing DTA and DSC results, some differences are noticeable. First, c-10Pd/Al_2_O_3_ (Fig. 8a) reveals a large standard deviation of the average DTA signal value of up to 12% at 450 °C, while DSC signal (Fig. 8b) exhibits only small standard deviation. Co_3_O_4_ and c-5Pd/Al_2_O_3_ shows the standard deviations within the measurement accuracy of the methods (although the deviation of c-5Pd/Al_2_O_3_ is in DSC noticeably higher). Second, the DTA response of c-10Pd/Al_2_O_3_ is quite high at 250 °C and increases continuously with temperature increase, while the DSC response is low at 250 °C compared to signals at higher temperatures and remains the same from 350 °C. The c-5Pd/Al_2_O_3_ shows also lower DSC response at 250 and 300 °C compared to DTA response. Third, the differences between individual values of the three catalysts are overall larger in DSC than in DTA despite the values at 250 °C what can be explained by higher sensitivity of DSC method and thus better differentiation of catalytic activity.

A large standard deviation reflects a low signal reproducibility, which originates from inhomogeneous distribution of Pd particles over alumina matrix which agrees with the SEM and EDX results (Fig. 3; Supplementary Information Figs. 1, 2). This is especially observed for a high amount of Pd in catalyst revealing higher inhomogeneity. The manufacturer also indicates the Pd content for c-10Pd/Al_2_O_3_ in a percentage range between 9 and 11%. In practice, the different local activity of catalysts is problematic and can lead amongst others to the occurrence of hot spots and thus to faster catalyst deactivation due to larger temperature gradients^44^.

The question arises why DSC measurements of c-10Pd/Al_2_O_3_ do not show such large standard deviation and no further response increase from 350 °C. Notably, DSC measurements were performed on the catalysts approximately 6 months after the end of DTA measurements. Short-term stability measurements help to explain these discrepancies. Thereby, the temperature dependent response of catalysts is measured bevor and after their treatment at 450 °C for 12 h and/or at 400 °C for 24 h in air.

**Figure 9:**
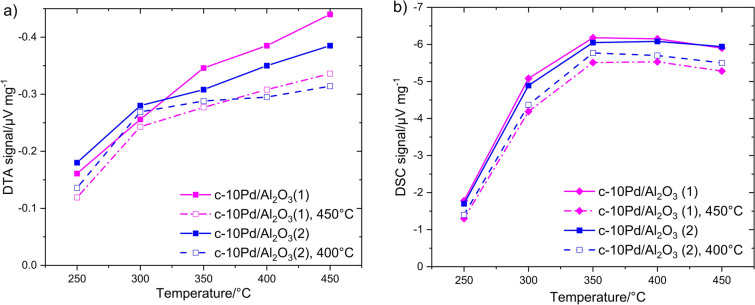
Temperature dependent (**a**) DTA and (**b**) DSC response of the commercial Pd/Al_2_O_3_ catalyst with 10 wt% Pd to 1% CH_4_ before (solid line) and after thermal treatment at 450 °C for 12 h (dash-dotted) and at 400 °C for 24 h (dashed line) in air.

**Figure 10:**
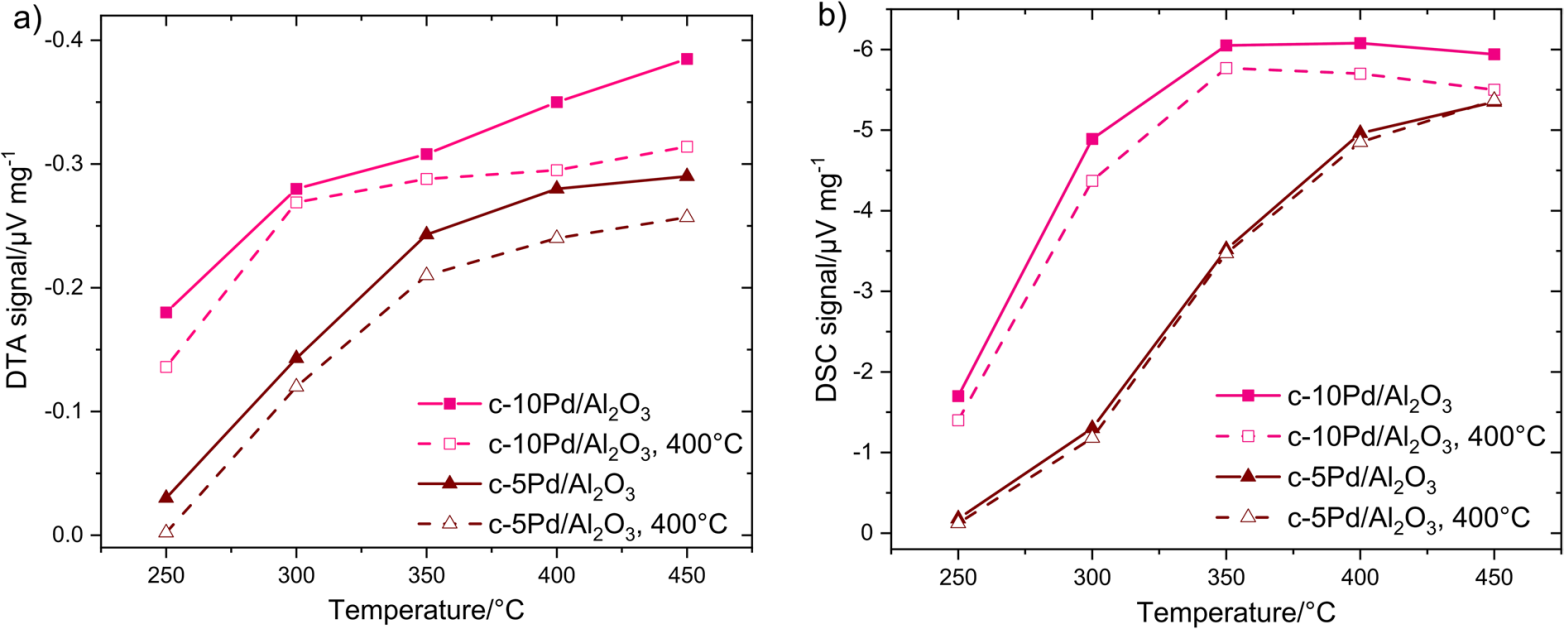
Temperature dependent (**a**) DTA and (**b**) DSC signal of two commercial Pd/Al_2_O_3_ catalysts with 10 wt% and 5 wt% Pd to 1% CH_4_ before (solid line) and after thermal treatment at 400 °C for 24 h (dashed line) in air.

According to DTA measurements (Fig. 9a), c-10Pd/Al_2_O_3_ has experienced considerable decrease in catalytic activity already after short treatment at both temperatures. Thereby, the response of treated catalysts increases negligibly with temperature rise from *T* ≥ 350 °C compared to the fresh catalysts. DSC (Fig. 9b) reveals smaller reduction of the response after thermal treatment. The same observations were made for c-5Pd/Al_2_O_3_ catalyst as shown in Fig. 10 for 400 °C in comparison with c-10Pd/Al_2_O_3_: strong deactivation of c-5Pd/Al_2_O_3_ for DTA (Fig. 10a) and hardly any effect of thermal treatment for DSC (Fig. 10b). The results state that both Pd/Al_2_O_3_ catalysts have lost part of their activity at the time of DSC investigations. This is possible because DTA and DSC measurements were performed with a time delay of 6 months. Co_3_O_4_ reveals no noticeable changes in the DTA or DSC signal after thermal treatment (not shown).

The fast deterioration of initial CH_4_ combustion activity of a Pd/Al_2_O_3_ catalyst at 350 °C has already been reported by Choudhary *et al.*^6^ Similar experience was made by Yang and Zhou testing Pt–Pd/Al_2_O_3_ for catalytic CH_4_ gas sensor at 400 °C^45^. DTA and DSC experiments evidence that thermal treatment in air strongly affects the activity of the investigated Pd/Al_2_O_3_ catalysts. The reason for that could be that initial activation^46^ of the c-10Pd/Al_2_O_3_ and c-5Pd/Al_2_O_3_ performed in a reducible atmosphere by provider is not sustainable^23^ and that numerous Pd particles cannot be sufficiently stabilized by the alumina support and lose their activity due to aggregation and/or other processes. The reoxidation of reduced Pd on Al_2_O_3_ is affirmed by change of the catalyst color from black to gray observed after the measurement. Different strategies have been reported to stabilize the particles of metal catalyst on the support material^47,48^.

## Conclusion

The present work demonstrates that DTA and DSC are convenient, easy-to-use methods for the fast estimation of the catalytic activity for CH_4_ combustion reaction, when simplified measurement procedure is applied. Thereby, the measurement of the baseline in air and the experimental measurement in test gas are carried out in one isothermal experiment, which excludes the effect of external thermal variations arising within the system.

It was shown by examination of CH_4_ (1 vol%) oxidation on a Co_3_O_4_ catalyst that both thermal analysis techniques can be used to characterize catalysts and to compare their activity while keeping the instrumental parameters influencing thermal signal constant. It was demonstrated that the vessel size, its geometry and the mass of catalyst used have a considerable impact on the mass-related DTA and DSC signals. In comparison to DTA, DSC measurements are more precise due to absence of mass transfer limitations inside the sample vessel and give more differentiated information about the catalytic activity. We see the advantage of DTA over DSC in the robustness of the sample carrier and crucible, which should enable tests with catalysts poisons or inhibitors. The corresponding experiments to test the poison susceptibility of catalysts are running. Another advantage of the method shown is the coupling of DTA with MS, which allows analysis of reaction products and additional insights into the reaction. For investigation of reactions with a low reaction heat or for higher signal differentiation, the use of small crucibles is preferred.

Finally, the potential of thermal analysis methods was illustrated using an application example. The catalytic behavior of Co_3_O_4_ was compared with two commercial Pd/Al_2_O_3_ catalysts containing 10 wt% and 5 wt% of Pd. Although Pd containing catalysts exhibit significantly higher activity and thus higher thermal response than Co_3_O_4_, they exhibit a low signal reproducibility, especially the catalyst with high Pd content, which was explained by a non-homogeneous distribution of Pd over alumina. Moreover, both Pd/Al_2_O_3_ catalysts show a rapid decrease of the initial thermal response under treatment in air at 400 °C (for 24 h) and 450 °C (for 12 h). The fast deterioration of methane oxidation activity of the Pd/Al_2_O_3_ catalysts was explained by poor stabilization of the Pd particles by the alumina matrix and the oxidation of initially reduced Pd.

## Supplementary Information

Supplementary Information.

## Data Availability

Data available on reasonable request from the corresponding author.
