# Supplementary material for: Differential thermal analysis techniques as a tool for preliminary examination of catalyst for combustion
Source: Sci Rep. 2023 Jun 16;13:9792. doi: 10.1038/s41598-023-36878-8 (PMC10276022; doi:10.1038/s41598-023-36878-8)
Supplement: Supplementary file 1 — Supplementary Information. [file 41598_2023_36878_MOESM1_ESM.pdf]

Supplementary Information

Differential Thermal Analysis Techniques as a tool for preliminary examination of catalyst for combustion

Olena Yurchenko[1,*], Hans-Fridtjof Pernau[1], Laura Engel[1,2], Jürgen Wöllenstein[1,2]

[1]Fraunhofer Institute for Physical Measurement Techniques IPM, Georges-Koehler-Allee 301, 79110 Freiburg, Germany
[2]Department of Microsystems Engineering - IMTEK, University of Freiburg, Georges-Koehler-Allee 102, 79110 Freiburg, Germany

*olena.yurchenko@ipm.fraunhofer.de

Using the EDX method, the weight percentage of each element present in the catalysts was studied.

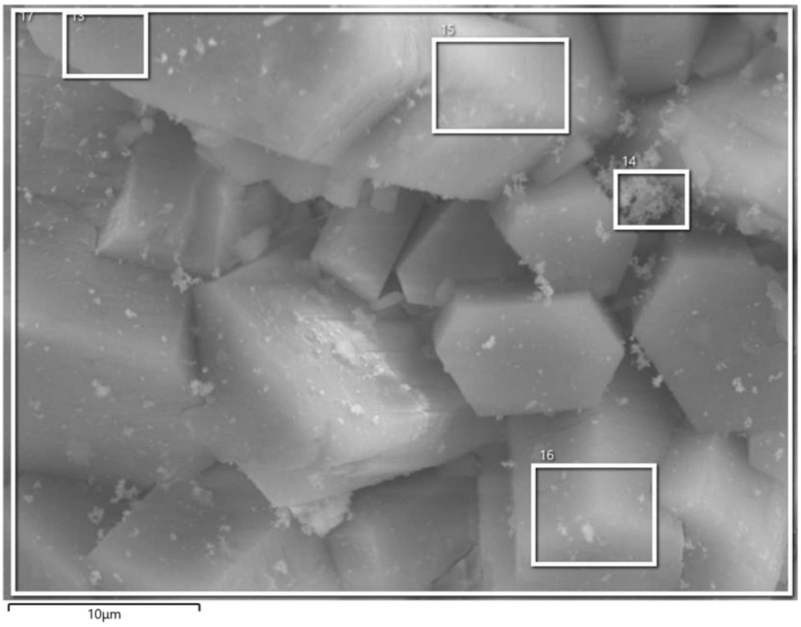

Figure 1. SEM image of c-5PdAl_2O_3 catalyst with depicted position of the recorded EDX spectra.

Spectrum	O wt%	Al wt%	Pd wt%
**13**	53.8	44.9	2.3
**14**	44.1	28.2	27.1
**15**	53.7	42.0	4.3
**16**	59.2	38.0	2.3
**17***	53.3	42.0	4.2
**Mean**	52.9	43.9	8.0
**Max**	59.7	28.8	27.0
**Min**	44.1	38.9	2.3
**Std deviation**	5.6	6.0	10.7

*overview spectrum

Table 1. EDX results for c-5PdAl_2O_3 catalyst.

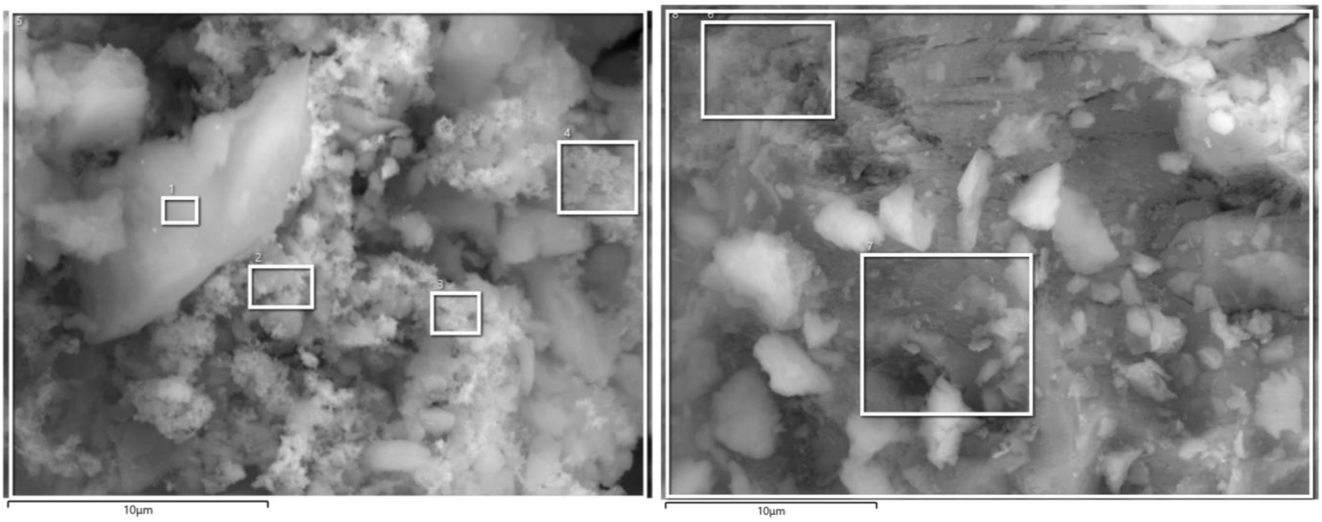

Figure 2. SEM images of c-10PdAl_2O_3 catalyst with depicted position of the recorded EDX spectra.

**Spectrum/ content**	**O wt%**	**Al wt%**	**Pd wt%**
**1**	49.0	39.9	11.1
**2**	27.7	26.8	45.5
**3**	29.1	20.2	50.7
**4**	31.4	24.1	44.5
**5***	37.1	28.7	34.2
**6**	27.1	52.9	20.1
**7**	33.4	51.9	14.7
**8***	38.2	49.0	12.9
**Mean**	37.9	36.7	29.2
**Max**	49.0	52.9	50.7
**Min**	27.7	20.2	11.1
**Std deviation**	8.3	13.4	16.4

*overview spectrum

Table 2. EDX results for c-10PdAl_2O_3 catalyst
